# Uncovering re-traumatization experiences of torture survivors in somatic health care: A qualitative systematic review

**DOI:** 10.1371/journal.pone.0246074

**Published:** 2021-02-04

**Authors:** Ana Carla S. P. Schippert, Ellen Karine Grov, Ann Kristin Bjørnnes

**Affiliations:** 1 Institute of Nursing and Health Promotion, Oslo Metropolitan University, Oslo, Norway; 2 Akershus University Hospital, Oslo, Norway; Erasmus Medical Center, NETHERLANDS

## Abstract

Little research has focused on torture survivors’ re-traumatization experiences in health and hospital units that treat somatic diseases, though any medical procedure can re-traumatize survivors. This study’s purpose was to summarize qualitative research evidence on torture survivors’ somatic healthcare experiences and to identify “triggers” or “reminders” that can lead to re-traumatization. The study’s search strategies identified 6,326 citations and eight studies, comprising data from 290 participants, exploring encounters with healthcare providers from torture survivors’ perspectives, which were included in the present research. Dallam’s Healthcare Retraumatization Model was used as a framework for data extraction and analysis. Five main themes were elicited from the findings: (1) *invisibility*, *silence*, *and mistrust*; (2) *healthcare providers’ attitudes and a lack of perceived quality in healthcare*; (3) *disempowerment*; (4) *avoidance*; and (5) *satisfaction and gratitude*. An analysis of the study’s findings revealed that torture survivors do not receive adequate healthcare and may experience challenges during treatment that can result in re-traumatization. The findings of this literature review provide a basis for understanding the difficulties that survivors experience in receiving somatic healthcare, as well as an explanation of the re-traumatization process.

## Introduction

Refugees and asylum seekers may face several challenges in adapting to a new country, and many have witnessed or experienced multiple traumatizing events that constitute exposure to torture [1–5]. The United Nations (UN) defines *torture* as “any act by which severe pain or suffering, whether physical or mental, is intentionally inflicted on a person … by or at the instigation of or with the consent or acquiescence of a public official or other person acting in an official capacity” [6]. According to Amnesty International in 2015–16, torture is practiced in over 140 countries worldwide, and torture survivors constitute a significant group among asylum seekers and refugees [2, 7–9], estimated at 5% to 35% [1]. Some studies have estimated torture survivors’ prevalence at up to 76% [10] among adult refugees and at approximately 11% [11, 12] among patients in various health and hospital units.

Sequelae after torture are manifold, and they represent a complex challenge for the healthcare system. Torture exposure is significantly associated with post-traumatic stress disorder (PTSD) [2, 3, 7, 13–17], and torture is considered one of the most traumatizing exposures, with a particularly high risk of developing mental health symptoms and, later, PTSD [6, 16–20]. Such mental health problems as memory disturbances, difficulty in concentrating, lack of energy, sexual dysfunction, emotional irritability, loss of trust, insomnia, nightmares, phobias [12, 21], depression [1], anxiety, and psychosis [18] are common among torture survivors. The most common somatic consequences of torture are neuropsychological pathology, broken bones, joint and muscle pain, headaches, dizziness, burns, and hearing loss [15, 19, 20, 22].

Most of the previous research on torture survivors has been conducted by psychiatrists and psychologists [23–27], while researchers involved in treating physical symptoms have paid less attention to torture survivors [3, 12, 22, 28]. This tendency is concerning since several of the sequelae after torture are physical (pain, deformities after fractures, weight loss, hypertension) and require treatment in health and hospital units that are specialized in treating physical disorders [29].

Healthcare providers are in a unique position to establish trust and relationships with patients, identify the potential signs and symptoms of torture, and listen to survivors who want to share their experiences. However, healthcare providers are often unfamiliar with torture survivors’ health problems [12, 30, 31]. This lack of knowledge, awareness, and training may affect patients directly, resulting in incorrect diagnoses, incorrect or inadequate treatments, and re-traumatization [4, 22]. *Re-traumatization* is the reactivation of trauma symptoms via thoughts, memories, or feelings related to the past torture experience. They can result from events or interactions in healthcare settings that remind survivors of their previous traumatic experiences.

Dallam’s Healthcare Retraumatization Model [32] was developed to help understand the difficulties that survivors are likely to face when accessing healthcare. The model is based on data from qualitative studies exploring childhood sexual abuse survivors’ healthcare experiences. It is the only model that explains the re-traumatization process based on a literature review, and it suggests that re-traumatization is a cyclical process with four interactive sub-processes: (1) hypersensitivity to threats to safety, (2) exposure to triggers, (3) post-traumatic stress reactions, and (4) avoidant coping [32]. An important aspect of the Healthcare Retraumatization Model is the concept of *triggers* [32]. Dallam (2010) described *sensory triggers* as stimuli in healthcare situations that resembled stimuli that were present at the time of abuse, and she described *relational triggers* as situations in healthcare that result in a sense of threat due to the relational dynamics between patients and healthcare providers. *Mixed triggers* are relational triggers that also include sensory components [32]. When experiencing re-traumatization, patients tend to avoid future healthcare. Re-traumatization occurs as a response to survivors’ exposure to triggers, and it activates strong emotional reactions and destructive coping attempts [32–34].

Torture survivors have been victims of brutal violence inflicted by a human being, and these experiences might have destroyed survivors’ trust in other humans [35]. Survivors can experience encounters with people in official positions—such as healthcare providers dressed in uniforms—as threatening, which may lead to re-traumatization [36]. Any kind of medical assessment or treatment has the potential to re-traumatize survivors of torture, and the fear associated with this process grows if a medical treatment is invasive or if it involves any similarities with a torture method [36, 37]. To increase safety and quality care for patients who have been victims of torture, healthcare providers must be familiar with potential stressors in order to adapt standard treatments [38]. Healthcare providers have documented challenges in providing effective and high-quality healthcare to torture survivors [16, 39, 40], and survivors have reported difficulties accessing healthcare, especially mental healthcare [41–43]. The literature has seldom focused on torture survivors’ re-traumatization experiences and the literature mentioning torture survivor’s re-traumatization in healthcare has been superficial without explaining the process of re-traumatization [44]. A consensus had been reached about medical procedures and medical equipment serving as triggers [12, 30, 38, 45–47], but the literature has presented conflicting data about the disclosure of torture or trauma stories as a trigger [4, 34]. Therefore, a synthesis of the literature based on torture survivors’ experiences in somatic healthcare may improve the understanding of necessary adaptations to standard healthcare treatments in order to prevent re-traumatization. The present literature review aims to summarize studies on torture survivors’ experiences in receiving treatment for somatic diseases in order to identify “triggers” and “reminders” in the healthcare context that can lead to re-traumatization.

## Methods

This study’s review protocol was guided by the *Standards for Systematic Reviews* by the Institute of Medicine and the PRISMA-P checklist [48–51] and registered on PROSPERO (International Prospective Register of Systematic Reviews). The PRISMA-P (A Preferred Reporting Items for Systematic Reviews and Meta-Analyses) checklist comprises 27 items designed to help authors improve their reporting in systematic reviews and meta-analyses [49]. The present review was limited to primary qualitative research studies that were available in several languages, which Table 1 shows, exploring encounters with healthcare providers from torture survivors’ perspectives. No date limits were imposed on any database.

**Table 1:** Eligibility criteria.

**Participants**	Torture survivors include refugees, asylum seekers, and war survivors. Adult participants (age >18 years old), of both sexes, are included
**Intervention**	Any somatic treatment administrated by health care providers working in health and hospital units intended to help refugees and asylum seekers who have experienced torture
**Context**	Somatic health care /medical care
	Specialist health care /hospitals
	Emergency service
	Intensive care units /critical care
	Outpatient clinics
	Primary health care /general practice /family practice
**Outcomes**	Experience
	Perception
	Feelings
	Expectations
	Critical episodes
	Obstacles
	Opportunities
	Patient-physician relation
	Patient acceptance
**Study design**	Qualitative

[A description of the concepts mentioned above is added to this manuscript as S1 Table.]

Since the PICO (patient, intervention, comparison, outcome) model is frequently used as a tool to structure clinical research questions in connection with evidence syntheses (e.g., systematic reviews) [52], we chose it as a tool for our search strategy. The PICO model is presented in Table 1.

### Search strategy

The systematic search for this review was conducted in August 2019, using the following databases: Cinahl, Cochrane Library, Embase, Medline, PsycINFO, PILOTS, Web of Science (ISI), OPENSIGLE, and WHO: International Clinical Trials Registry Platform /ICTRP. Since relevant material is often published by torture survivor centers or other gray-literature sources, a scan of relevant websites was included in this process. The PICO framework was used to develop the search terms relevant to the PICO question [52]. Medical Subject Headings (MESH) with related text-based search terms were used with a combination of terms and concepts, in accordance with databases’ concordance [53, 54]. Citations from relevant articles and systematic reviews were also screened. A full description of the search strategy is provided in S2 Table.

**Fig 1:**
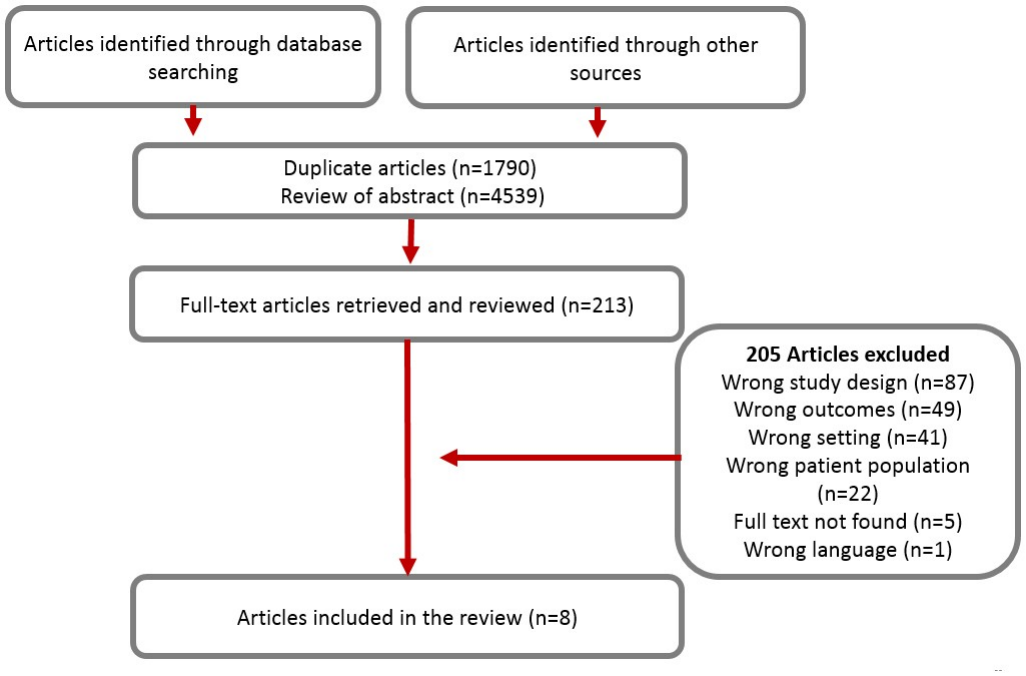
Search strategy and article review process.

This study’s search strategies yielded 6,329 citations, which were imported into the Covidence literature-screening software [55], and 1,790 duplicates were removed. Two independent reviewers (ACS and AKB) assessed 4,539 titles and abstracts against the eligible criteria. Any disagreement was resolved by consulting a third reviewer (EKG) until reaching a consensus. A total of 213 full-text articles met our inclusion criteria and were retrieved. Eight studies were included in the final review. Fig 1 presents the search strategy and the article review process.

The eight included studies represented six countries, were published between 1993 and 2018, and comprised data from 290 participants. Their data collection was based on semi-structured, in-depth interviews [56–59], and focus groups [60, 61]. One study used a case study approach [62], and one study used storytelling as a data collection method [63]. All the studies included in this review were conducted in high-income countries (i.e., Canada, England, the United States, Denmark, Switzerland, and Ireland) and presented refugees and asylum seekers’ healthcare experiences.

### Data extraction and synthesis

Two researchers were involved in the data extraction process (ACS and AKB). A summary of evidence was processed for each study. Dallam’s Healthcare Retraumatization Model [32] was chosen to create a framework for our data synthesis, and the model’s four sub-processes were used to create a framework of our identified themes.

Data from the included studies were coded toward the thematic framework, and line-by-line coding relating to refugees’ experiences provided the descriptive themes. We interpreted data that could not be included in the framework using interpretative and inductive analyses [64]. Using inductive analysis, we moved from specific data to a general theoretical understanding. An extensive text was condensed into a summary of findings, and in this process, we established links between our findings and our research objectives [64]. Two authors read this text in detail, familiarized themselves with its content, and gained an understanding of the themes covered in the text. The authors identified and defined sub-themes and themes. The sub-themes were derived from multiple readings of the raw data. Using inductive coding, the sub-themes were created from actual phrases and meanings in specific text segments. As a result of this analysis, we developed categories as a framework that summarized the raw data and conveyed key sub-themes. Different and overlapping descriptive sub-themes were noted and summarized.

To improve the overview and to ensure a comparison of the findings with Dallam’s Healthcare Retraumatization Model [32], we grouped the sub-themes into main themes. This deductive approach ensured an ability to benefit from an existing model, and our inductive analysis ensured an ability to develop a model that could explain the re-traumatization process of torture survivors based on the underlying structure of their treatment experiences for somatic conditions. This process is illustrated in S1 Fig. Some of the sub-themes could have been simultaneously included in more than one of the themes; however, to create a clear and highly rigorous system, we placed each sub-theme into only one theme. Although our findings could have been grouped differently, we found the entities in the units we established to be appropriate for adaptation using Dallam’s Healthcare Retraumatization Model. The data extraction and synthesis processes are illustrated in S1 Fig.

### Assessment of methodological quality

Appraising different qualitative studies in relation to the specific methodology used in research is recommended because researchers’ chosen approach is linked to the “outcome” of their research [65]. Therefore, a quality assessment of our included studies was performed using the Joanna Briggs Institute (JBI) Standardized Critical Appraisal Checklist for Qualitative Research [66]. This tool evaluates studies’ methodological quality according to a 10-object scale, accounting for coherence between philosophical perspectives, data collection methods, and qualitative analysis—including studies’ representativeness of participants and their voices, ethical criteria, and researchers’ influence on interpreting the research (S3 Table). The purpose of this appraisal is to assess a qualitative study’s methodological quality.

Each study in the present review was independently assessed for quality by two reviewer authors (ACS and AKB), and disagreements were resolved by referring to a third review author (EKG). The summary of our findings also includes an overall assessment of the findings’ confidence, according to the GRADE-CERQual (Confidence in the Evidence from Reviews of Qualitative Research) [67, 68] to assess how much confidence should be placed in findings from the data synthesis, based on four components: methodological limitations, the included studies’ relevance, the data’s coherence, and the data’s adequacy. An assessment based on judgments of each of the four CERQual components was summarized at four levels: *high*, *moderate*, *low*, or *very low*. This assessment is presented in S4 Table.

### Synthesis of findings

Table 2 outlines the included studies’ characteristics and the healthcare experiences presented in each study.

**Table 2:** Descriptive information for the included studies.

Author (year), country: title	Design	Aim	Study methods	Sample	Country of origin	Services explored	Experiences of health care
**Berman et al., (2006), Canada: A narrative Study of refugee Women Who Have Experienced Violence in the Context of War**	Interpretative study	To examine the health care experiences of refugee women who experienced violence in the context of war	Individual semi-structured interviews; One individual interview with a duration of two hours; A focus group with six of the participants	9 refugee women	Bosnia; Guatemala; El Salvador; Chile	General health care; Prenatal services	Health care providers´ lack of understanding and interest; Hostility; Racism; Violence; Sexual assaults by physicians; Sense of violation; Shame and anger under treatment
**Fang et al., (2015), England: Experiencing ´pathologized presence and normalized absence´; understanding health related experiences and access to health care among Iraqi and Somali asylum seekers, refugees and persons without legal status**	Exploratory study	To explore on-going health problems and challenges when seeking treatment and care	35 in-depth interviews and five focus groups	66 asylum seekers, refugees and persons without legal status. Men and woman.	Somalia; Iraq	General health care services	Waiting time; Health care; Professionals are always busy; Short consultations; Even shorter consultations with translators; No time to generate trust
**Gruber & Byrd, (1999), USA: Post-Traumatic Stress Disorder and GI Endoscopy: A case Study**	Phenomenological study	To offer suggestions to health care professionals and endoscopy nursing staff who care for patients who survived torture	A case study	1 man, torture survivor, captured and imprisoned by the Vietnamese under war	USA	Endoscopy services	Flashbacks; Deathly afraid; Worried that the procedure would trigger flashbacks and violent behavior; Relaxation stimulated to verbalize horrors from war; Disoriented to place;
**Hermansen & Nielsen, (2018), Denmark: Betingelser I hverdagslivet i eksil, som kan få betydning for mødet med det danske sundhedsvæsen—et kvalitativt studie (Conditions in everyday life in exile, which may have an impact on the meeting with the Danish healthcare system—a qualitative study)**	Phenomenological Hermeneutic study	Exploring perspectives on exile and meeting with the Danish health care system	Individual semi- structured interviews	5 refugee women	Iraq; Lebanon	Danish University Hospital	Not believed when communicating pain; Pain and stress not recognized and not treated; Palpitations when sent to a health control
**Perron & Hudelson, (2006), Switzerland: Somatization: illness perspectives of asylum seeker and refugee patients from the former country of Yugoslavia**	Exploratory study	To understand how somatization, make sense of asylum seeker and refugee patients suffering. To explore health care expectations	Semi-structured interviews	26 asylum seeker and refugee 14 from Kosovo 11 from Bosnia-Herzegovina and 1 from South Serbia	Yugoslavia	General medicine outpatient clinic of a Swiss teaching hospital	Openness; Respect; Understanding; Interest; Listened to; Don’t like appointments with doctors; Don’t like to talk about the past; Short consultations; Poor communication; Fear of being labeled as mentally ill; Discrimination because of legal status
**Shannon, O’Dougherty, & Mehta, (2012), USA: Refugees’ perspectives on barriers to communication about trauma histories in primary care**	Exploratory study	To explore refugees`perspectives regarding the nature of communication barriers that impede the exploration of trauma histories in primary care	Interviews	50 refugees women and men	Liberia; Laos; Thailand; Vietnam; Cambodia; Nigeria; Kenya; Ethiopia; Ivory Coast: Bosnia; Peru; Colombia	Suburban primary care clinic	The doctor did not raise the topic; Not the purpose of the clinic visit; Patients don’t want to talk about it/don’t want to remember; Patients want to move on; Language as a barrier; Cultural barriers; Time as a barrier
**Shannon P.J, (2014), USA: Refugees’ advice to physicians: how to ask about mental health**	Exploratory study	Refugees offer advice to physicians about how to assess the mental health effects of trauma based on their experiences of health care	Focus Groups	111 refugees women and men	Burma; Bhutan; Somalia; Ethiopia	General health care	Short appointments and change of interpreters as a problem; Multiple providers; Lack of trust; Cultural barriers
**Tobin, Murphy-Lawless, & Beck, (2014), Ireland: Childbirth in exile: Asylum seeking women’s experience of childbirth in Ireland**	Exploratory study	To gain insight into women´s experiences of childbirth in health care institutions while in the process of seeking asylum	Narratives	22 asylum seekers women	Nigeria; Cameron; Burundi; South Africa; Zimbabwe; Iran; Iraq; Zaire; Sierra Leone	Maternity department	Lack of connection, communication and cultural understanding; Language as a barrier; Distress; Trauma; Discrimination; Racism; Inappropriate care

**Fig 2:**
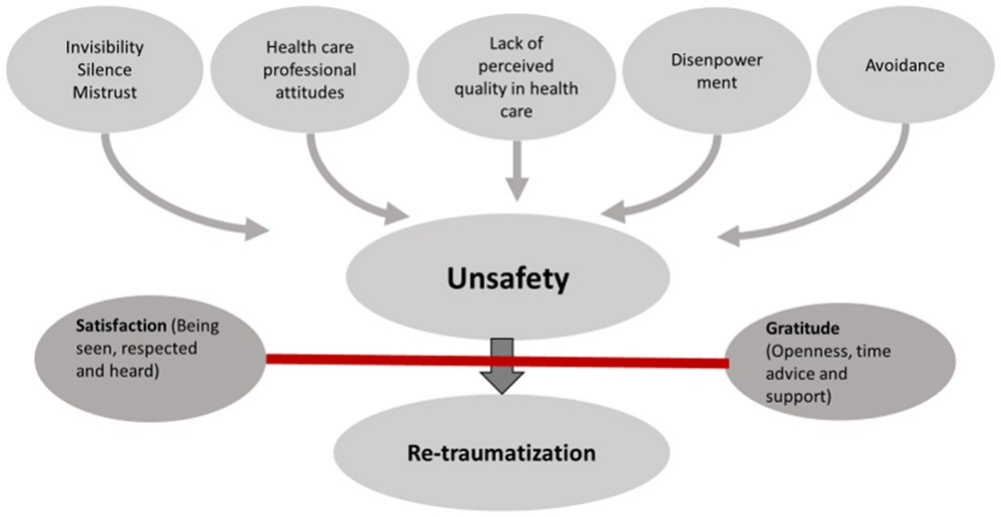
Explanatory model for the experiences of refugees and asylum seekers that can cause re-traumatization in somatic health care.

Five main themes were elicited from our findings: (1) *invisibility*, *silence*, *and mistrust*; (2) *healthcare providers’ attitudes and a lack of perceived quality in healthcare*; (3) *disempowerment*; (4) *avoidance*; and (5) *satisfaction and gratitude*. To develop a healthcare re-traumatization model for torture survivors, based on an existing model, we adapted four of the grouped findings to the four interactive sub-processes of Dallam’s Healthcare Retraumatization Model: “hypersensitivity to threats to safety,” “exposure to triggers,” “post-traumatic reactions,” and “avoidant coping” [32]. Table 3 shows the grouped findings and their adaptation to the Dallam’s Healthcare Retraumatization Model concepts. Subsequently, the themes were used to create a re-traumatization model for torture survivors in healthcare via a comparison with the Dallam’s Healthcare Retraumatization Model. The fifth group of findings, *satisfaction and gratitude*, is not framed in the healthcare re-traumatization model since it represents resources for avoiding re-traumatization. We discuss all of our findings below and use them to create an explanatory model for refugees and asylum seekers’ experiences that can cause re-traumatization in somatic healthcare, which Fig 2 presents.

**Table 3:** Grouped findings in themes and their adaptation to Dallam’s Healthcare Retraumatization Model.

Themes identified	→	Adaptation of themes and sub-themes to the Health Care Re-traumatization Model
Invisibility	**→**	
Silence		Hypersensitivity to threats to safety
Mistrust		
Health care providers’ attitudes	**→**	Exposure to triggers
Lack of perceived quality in health care		
Disempowerment	**→**	Post-traumatic reactions
Avoidance	**→**	Avoidant coping
Satisfaction and gratitude		

### Negative experiences: Contributing factors in re-traumatization

#### Invisibility, silence, and mistrust

*The invisibility*, *silence*, *and mistrust* theme includes findings that indicated a strong need to be seen as a person with a story including several traumas. Torture survivors showed an increased dependence on healthcare providers and a severe lack of trust in others. This group of findings also included a hypersensitivity associated with the past and refugees’ or asylum seekers’ legal status. One participant expressed this hypersensitivity by stating, “I was destroyed as a result of the past, and then the illness made me worse” [58].

Survivors expressed that their legal status as refugees tended to limit their rights to healthcare, causing feelings of invisibility. They also expressed concern about the possibility of being returned to their home countries and a hope that healthcare providers could help them get legal residency status. These feelings seemed to cause a form of dependence on healthcare providers for survivors’ recovery from their past lives and suffering [56, 58].

A refugee from the former country of Yugoslavia expected that her physician would help her resolve her social problems, saying, “I don’t want to be expelled, I don’t want to suffer anymore. … I have two friends, they got the B permit (legal status) and say that they got it through their physician” [58].

The patient’s expectations that healthcare providers would understand their stories as refugees and help them not only with health challenges and symptoms but also with social problems exceeded healthcare providers’ real offers of help. Patients may feel disappointed and invisible when healthcare providers do not address these needs [56].

A participant in a study examining everyday life in exile expressed disappointment with healthcare providers: “Some don’t want to know the story and they don’t have time to listen” [59]. In the same study, a participant expressed disappointment with healthcare: “When I come to the hospital, I want to have more tests done or at least be hospitalized to do everything, to find out what I have. I asked my doctor, but maybe because I am a refugee, they don’t do that” [58].

When expectations are not met, patients became disappointed and lost hope. A participant verbalized this effect by saying that “she never wants to have another child” [63] in order to avoid being hospitalized again. These feelings also reinforce mistrust.

Patients expected genuine interest when asked about their scars and past lives but felt that healthcare providers often asked about these topics only for the sake of conversation but did not care. This perspective may cause great disappointment for survivors of war and torture, making them feel that their stories and special situations are met with silence. This silence caused feelings of invisibility and a desire to share experiences [60].

A survivor responding to an interview conducted at a suburban clinic expressed expectations for healthcare providers to show more interest in the history behind physical scars: “When he saw the scar on my (part of the body) he asked me what happened and how I got it. We didn’t go into detail about it and I didn’t talk of other things that happened in the war” [59].

In a study about barriers to communication about trauma histories, a refugee expressed an expectation that her physician would take initiative to ask about her traumatic war experiences: “I feel I should be asked before I bring anything up. It’s hard to just start talking about these things to your doctor” [59].

In addition to basic trust in others having been possibly been destroyed by traumatizing events of war, flight, and torture, survivors’ disappointment with healthcare providers, their feelings of invisibility, and the silence surrounding their stories reinforced their mistrust in healthcare providers during their healthcare encounters [58].

#### Healthcare providers’ negative attitudes and a lack of perceived quality in healthcare

The *healthcare providers’ negative attitudes and a lack of perceived quality in healthcare* topic summarized findings that were largely connected to healthcare providers’ behavior and attitudes. Traumatized survivors of war and torture perceived that healthcare providers sometimes acted unfriendly, expressing discriminative attitudes like racism and even hostility [56, 60]. Some participants suggested that healthcare professionals had made them feel that they did not deserve care [58]. Participants in a study that included refugees attending a general outpatient clinic believed that they did not deserve care because of their refugee status [60]. Confronting such attitudes made survivors feel uncomfortable, preventing the development of trust and destroying possibilities of communication. Feelings of being discriminated against because of their race intensified when participants interacted with healthcare providers of different races than their own. Participants in a study analyzing experiences of childbirth in exile expressed this view; one participant described feelings of discomfort and fear during birth at a hospital: “I was the only black there, so I was so scared I didn’t want to make them, to put them off” [63].

Survivors of war and torture face an unfamiliar environment and perceive many differences in their host country’s healthcare. They experience a lack of understanding from healthcare providers, regarding both cultural aspects and the complex circumstances of being a survivor [56, 63]. In a study presenting refugees’ advice to healthcare providers, a participant expressed the importance of cultural understanding, saying, “It may be easier to trust the process if someone from their own cultural background is there helping to ask the questions” [60].

Participants also experienced healthcare providers not taking an interest in learning about refugees despite their low level of knowledge [56]. Survivors felt rejected when healthcare personnel were dismissive of their culture, experiencing tactile stimuli under medical examinations as an insult and lacking trust in healthcare providers, which presented a barrier to discussing intimate details or complex health issues [59–61, 63]. Participants expressed a preference for healthcare providers of their own backgrounds [60].

Survivors also experienced a lack of trust among healthcare providers and a failure to provide satisfactory treatment when they presented with symptoms of pain [57]. In a study that examined encounters between female refugees from the Middle East and healthcare providers, all the participants described their pain as having not been taken seriously [57]. One participant remarked, “For example, if I tell my doctor that I have a real pain in my head, he says ‘Panodil’ or sometimes he takes his book and writes Panodil. Is it enough? No if I’m a patient and in pain, find out why. You need to examine me” [57].

Torture survivors often expressed difficulty in building trust through contact with healthcare providers and interpreters, especially because of a lack of time [58–61]. One participant in a study that gave advice to healthcare providers explained, “It takes time to build a relationship and get comfortable with interpreters and doctors” [60]. In a study that included Iraqi and Somali asylum seekers, refugees, and people without legal status, one participant expressed a lack of time as a barrier: “They [GPs] have their own style of examining and there, err, there can be such patients that you cannot examine properly in the limited time the GP has his fixed intention of making 5 min, 10 min, he won’t have had much time to assess, to go in depth in cultural situation” [61].

Survivors also reported negative experiences with the quality of healthcare services because of they felt that they received inappropriate care and challenges in communicating with staff. This perspective was clearly expressed by participants in a study analyzing experiences of childbirth in exile [63].

#### Disempowerment

Patients can feel powerless because of the imbalance that characterizes the relationship between patients and healthcare providers as authority figures [36]. Survivors mentioned strong and negative feelings under treatment, and the *disempowerment* theme was prominent when care related to—for example—gynecological examinations [56, 63]. They expressed feelings of fear, distress, suffering, and violation, and they reacted with submission, shame, and anger [56, 63]. A study exploring childbirth experiences found that refugees were cautious in showing disappointment because they feared punishment [63]. A participant described her experiences of childbirth at an Irish hospital: “I don’t want to speak; I thought they would beat me the same way if I scream or I cry. So, in labor I don’t speak, so that I don’t upset them” [63]. Thus, a lack of language fluency, combined with a fear of mistreatment, resulted in a survivor’s total silence. This participant also clearly expressed a feeling of disempowerment when she said that “dying would have been better than going through all these things alone” [63].

Feelings of overwhelming stress, and even flashbacks, were mentioned especially when medical equipment was used [62, 63]. Experiences of disorientation concerning place and time in recovery were connected with the use of anesthetics, causing feelings of disempowerment.

Survivors also reported being triggered by healthcare providers using specific words and phrases similar to words and phrases their abusers had used, which led to feelings of great fear. One study participant recounting her experiences with the Danish healthcare system expressing feelings of stress as a consequence of the terminology healthcare providers had used: “My doctor said—You go to a control—But I didn’t understand a health control. If you say—control—in my home country, that means something big. I was thinking, uha control to me? When I came to the health center to control my heart started to beat” [57].

Survivors also experienced language difference challenges in clinical settings [59–61, 63]. They expressed feelings of fear and distress when they did not understand healthcare providers [63]. Because they did not understand healthcare providers’ language, survivors felt disempowered and were always afraid that they would receive inappropriate treatment that would put their lives at risk [63].

Patients also reported that the use of interpreters made appointments seem shorter, which reduced their perceived quality of care [61]. Challenges in building trust with interpreters were also expressed, especially when interpreters were changed frequently. A participant in a study that included refugees from Burma, Bhutan, Somalia, and Ethiopia explained that “it takes time to build a relationship and get comfortable with interpreters and doctors” [60] and suggested that healthcare providers and interpreters should take this time.

Telling their stories of war, fleeing, and torture to healthcare providers seemed important to patients [59, 60], but they struggled with language barriers in sharing trauma histories [60], referring to the need for interpreters. Moreover, they reported that changes to their interpreters caused dissatisfaction and contributed to a lack of trust in their interpreters [60]. Although some survivors regretted not being asked about their stories by healthcare providers, others reported that telling their stories raised bad memories.

#### Avoidance

*Avoidance* of appointments with doctors and avoiding disclosure was another frequently reported theme across the included studies. A lack of trust in healthcare providers and interpreters was generally why survivors did not disclose their histories in healthcare encounters. In a narrative study of refugee women who had experienced violence, one participant explained why she had avoided disclosing her history: “I am afraid of talking or disclosing to medical doctors for fear of having a medical record that later could be used against me” [56].

Survivors of war and torture reported that some healthcare providers did not understand their past trauma [56], and they felt mentally labeled by healthcare providers [58]. This perspective caused distress and was also a reason for withholding trauma histories [58]. Other reasons for survivors choosing not to tell their stories to healthcare providers included a perception that healthcare providers neither had the time to listen to these stories nor the competence to ask about them. A refugee who responded to questions about whether and how physicians should ask about traumatic experiences responded, “No, neither my doctor nor my baby’s doctor has asked about the situation” [60].

Survivors expressed feeling sick for days after telling their story to healthcare providers [59, 60]. Sometimes patients did not want to talk about these histories because they did not want to remember the past in order to move forward in their lives [59, 60].

### Positive experiences: Protective factors against re-traumatization

#### Satisfaction and gratitude

Survivors of war and torture accept health services when they feel seen by healthcare providers. When healthcare providers showed respect and interest, meeting survivors with openness and time, survivors reported feeling great satisfaction [58]. Feeling supported was mentioned as an important factor in resolving survivors’ isolation and lack of social networks. Survivors also reported that healthcare providers gave them the strength to continue by being kind, listening and offering advice [58, 63]. In one study, survivors reported positive experiences with healthcare providers, including gratitude for their care and a positive attitude among healthcare personnel [58]. Careful listening and words of comfort from nurses were mentioned as interventions to address survivors’ healthcare needs and neutralize feelings of fear during treatment [62].

These findings reflected various themes, and Fig 2 provides a visual summary of how these findings related to each other and could cause re-traumatization. Findings related to *satisfaction and gratitude* were protective factors, which could mitigate or eliminate the risk for re-traumatization in somatic healthcare.

### Quality appraisal

Our judgments across the 10 items of the JBI risk-of-bias tool for each individual study are presented in S3 Table. Across all included studies, we found partialities related to internal validity, and the results of the methodological quality assessment are outlined in the table.

Our overall CERQUAL assessment of confidence was graded as *moderate* because of moderate concerns regarding methodological quality, relevance, and coherence (S4 Table).

## Discussion

The findings of this qualitative systematic review confirm that survivors have expressed negative healthcare experiences. These experiences include feelings of invisibility, silence, mistrust, negative attitudes among healthcare providers, a lack of quality healthcare, disempowerment, and avoidance. Survivors also expressed positive healthcare experiences of satisfaction and gratitude.

These findings suggest that traumatized survivors of war and torture face challenges during their encounters with healthcare providers treating somatic conditions. In line with other literature, this review’s findings reveal that—though refugees and asylum seekers are removed from the immediate threat of torture—the psychological and physical impacts of war, flight, and torture can persist for a long time [22]. Despite poor past healthcare experiences, because of health problems, survivors need to seek healthcare help [62]. Our findings reflect some ambiguity, however. On the one hand, survivors do not want to seek healthcare help, and on the other hand, their genuine healthcare needs forced them to seek help [62]. According to Dallam, seeking help and entering the healthcare system can begin the re-traumatization process for survivors [32].

In adapting this review’s findings to Dallam’s Healthcare Retraumatization Model, we consider the theme *invisibility*, *silence*, *and mistrust* to compound Dallam’s factors that cause “hypersensitivity to threats to safety.” Meanwhile, we regard *healthcare professionals’ attitudes and a lack of quality healthcare* as equivalent to Dallam’s “triggers,” while *disempowerment* increases “post-traumatic reactions” and *avoidance* corresponds to “avoidant coping,” as Table 3 shows.

### Invisibility, silence, and mistrust as hypersensitivity to threats

Participants described feelings of not being heard or seen, causing disappointment and reinforcing mistrust. As Behnia described [35]—and in line with our findings—even many years after trauma, survivors experience the world as a dangerous place where people in positions of authority and caring roles, such as doctors, nurses, and interpreters, cannot be trusted [35, 60, 61, 63]. Consequently, survivors may perceive interactions and situations during treatment as threats to their safety [56, 63]. In a case Jennings [33] described about how victims of sexual abuse become re-traumatized through invisibility in healthcare, we see similarities with the current review’s findings on healthcare providers not considering survivors’ special needs or actual stories as important, which may make survivors feel invisible. Our findings suggest that feelings of fear and insecurity caused by threats during war, flight, and torture cause a hypersensitivity that emerges in interactions with healthcare providers. Factors that reinforce feelings of insecurity include survivors’ experiences of lacking trust and recognition among healthcare providers. We can adapt these findings to Dallam’s Healthcare Retraumatization Model [32], which describes *hypersensitivity to threats to safety* as the main factor determining survivors’ re-traumatization. Our findings also find support in a case study by Jacobs and Iacobino [36], which described how distrust makes survivors view interactions and healthcare settings as threatening [36]. Our findings indicate that survivors of war and torture expect to be met with understanding and acceptance among healthcare providers and to receive help in turning their vulnerability into a resource for receiving desired healthcare and social care [56, 58].

### Healthcare providers’ negative attitudes and a lack of quality healthcare as triggers

Several of our findings described aspects that healthcare survivors found triggering. When they feel that they did not have the same access to care or deserve the same attention as other people [58, 63], survivors may develop feelings of discrimination [69].

According to Dallam [32] and Hokland’s [34] descriptions of how triggers work, these feelings can trigger associations, dynamics, and memories about aggressors during war and torture, which may lead to re-traumatization. When survivors enter healthcare settings, they are automatically exposed to numerous challenges that can work as triggers [32]. This view supports our findings of several challenges that survivors have identified, including healthcare providers’ disinterested attitudes, which serve as triggers. Other studies have connected similar challenges to refugees’ healthcare encounters [70, 71]. According to Richey [72], healthcare providers may hesitate to involve themselves with survivors because of inexperience with this patient group and the complex problems they present. Such sensorial triggers as medical equipment [62, 63], waiting times, short consultations [58–61], language problems [59, 60, 63], and tactile stimuli [56, 58, 63] that were documented in this review have also been described by other authors [30, 73].

As other studies have suggested, and in line with our findings, trauma survivors perceive more challenges in interacting with healthcare personnel than other patients [74, 75]. Therefore, such mixed triggers as medical examinations [58, 63], tactile stimuli [56, 58, 63], and a lack of privacy—which combine both sensory and relational triggers [32]—may strongly affect survivors. These factors are usually perceived as part of health examinations or healthcare settings; however, for people who have experienced trauma, these factors can act as triggers, causing distress and concerns about receiving inappropriate care [56–61, 63].

Our findings reveal that survivors experience pain as a common health problem, which health personnel do not handle as survivors expected. According to a recent study about Islamic State (IS)- traumatized female refugees, pain is a major symptom among survivors [40]. Survivors have expressed that healthcare providers do not take their pain seriously, causing dissatisfaction and a sense that survivors are receiving low-quality healthcare. These feelings may also serve as triggers and cause potential re-traumatization.

Triggers may overlap with each other, be related to each other, and have multiple effects. Sensory triggers can cause distress because they resemble stimuli that were present at the time of torture, as Dehghan [76] described in a review on the health impact of sexual torture among Iranian and Afghan refugees. These stimuli can involve any kind of bodily sensations or sights, sounds, and smells in a treatment environment. In line with the Dallam’s Healthcare Retraumatization Model, we also found that triggers can result in physical reactions, such as shaking, nausea, or vomiting [63].

Among other findings in this review, survivors’ sense of not being believed when providing information about their situations to healthcare providers during treatment may remind them of interrogation situations, where victims are tortured because they are suspected, distrusted, falsely accused, or forced to confess [29]. When survivors interact with healthcare providers, these associations may cause re-traumatization.

A lack of cultural understanding—also called “ethnocultural discordance” [29]—was reported by asylum seekers in a Norwegian study [77] as one of the most important factors affecting outcomes in survivors’ healthcare. Our findings echo this view since a lack of cultural understanding was one of the triggers our review identified [56, 63]. According to our findings, both the use and eschewal of interpreters serve as potential triggers in the re-traumatization process [59, 60, 63]. A recent study that included Ethiopian asylum seekers in Norway [77] supports this finding, having reported that survivors felt a sense of autonomy and control when they expressed themselves but not when an interpreter spoke for them. Recounting traumatic war incidents is difficult, but it is even more challenging when such communication must be facilitated by an interpreter [78]. Accordingly, our findings suggest that such relational triggers as the imbalance between healthcare providers or interpreters and patients contribute to most of the triggering situations in healthcare. However, the addition of other triggers from other categories—such as a lack of privacy, medical equipment, sounds and smells in the treatment environment, and tactile stimuli during treatment—can intensify survivors’ distress, causing re-traumatization.

### Disempowerment causing post-traumatic reactions

As Dallam [32] described, reactions of discomfort during treatment disempower survivors and reinforce PTSD symptoms associated with re-traumatization. According to our findings in this review, survivors are triggered by experiencing disempowerment and physical reactions similar to what they experienced during their original trauma [62, 63]. Survivors can experience flashbacks triggered by aspects of encounters with healthcare personnel. Such flashbacks cause distress and difficulties in handling life [72]. Our findings also include other physical reactions—such as feeling sick, nausea, shaking, disorientation concerning place and time, and uncomfortable reactions to anesthetics, including feelings of losing control. Survivors also expressed concerns about accepting medications [62]. This finding is consistent with recent research about IS survivors’ concerns with taking any medications because they could remind survivors of sedation and immobilization during captivity, thus evoking feelings of disempowerment [40].

The power imbalance between healthcare providers and patients may also replicate the power imbalance between torture survivors and torturers, reinforcing other triggering situations in healthcare. Consequently, and in line with this review’s findings, survivors may become hypervigilant during treatment [62, 63]. Feelings of such power imbalances have also been described in studies about the re-traumatization of sexually abused women [79, 80], which revealed that such dynamics may cause victims to experience health examinations and touching as sexual assaults, evoking a sense of violation [56, 63], anger, and shame [56]. These reactions may make patients struggle fiercely against feelings of losing control, which can manifest as impatience, violent behavior, or silence [63]. These reactions indicate a high degree of disempowerment, which might lead to ethical concerns about obtaining informed consent from patients who are torture survivors.

### Avoidance

Our findings show that survivors experience several barriers to disclosing their stories [59, 60]. In line with this finding, Eisenman [12] revealed in a study that torture survivors may not volunteer to recount their torture histories due to feelings of guilt, shame, or generalized mistrust. In addition, healthcare providers often fail to identify histories of torture—even when patients believe torture has affected their health [12]. Moreno and Grodin [4] concluded that asking about torture should be part of interviews when caring for survivors. Our findings in this review indicate that approaches to caring for torture survivors should be evidence-based and linked to interview guidelines.

To overcome such barriers, survivors employ strategies of proactive coping [62, 63] to alleviate the effects of stress reactions during triggering. However, some survivors in the studies we examined [56, 63] ultimately avoided healthcare (i.e., avoidant coping) [32], avoided appointments with doctors [59], and stopped telling their stories to healthcare providers [59, 60]. Dallam [32] described avoidant coping’s ability to impede the development of trusting relationships with healthcare providers, interfering with survivors’ ability to seek essential care in order to maintain or improve their health. Torture survivors can regard healthcare providers as frightening because they are afraid healthcare providers might have connections to governments [36]. Concerningly, the coping strategies identified in this review—such as choosing not to seek treatment for serious illnesses, refusing treatments, failing to engage in treatment regimens, and abandoning treatment despite medical advice—can deteriorate survivors’ health and even lead to death.

### Satisfaction and gratitude as preventive factors against re-traumatization

Our findings suggest that being seen and listened to, being met with respect and openness, and receiving advice and support are what survivors ask for and expect from healthcare providers [58, 63]. These feelings of satisfaction and gratitude may prevent the re-traumatization process and remedy feelings of unsafety and invisibility. These findings expand on Dallam’s Healthcare Retraumatization Model [32] by including feelings of satisfaction and gratitude as factors that can prevent re-traumatization.

### Limitations and strengths

Although this review involved a limitation in the low number of available studies to assess, the included studies that we synthesized represented the experiences of 290 participants with different backgrounds and countries. We found similarities in participants’ concerns and experiences related to healthcare somatic services, which increase the review findings’ credibility. To ensure rigor, a prespecified plan was followed, and the data were rich and reasonably coherent across studies. One strength of the method we used is the possibility to condense extensive and varied raw text-data into a brief, summary format in order to establish clear links between the research objectives and the summary findings derived from the text data. This advantage made the data more available and transparent and, therefore, easier to understand for readers.

Our findings may have been shaped by our assumptions and experiences as researchers in deciding what aspects of the data were more important and less important. Another issue we must address is that inductive analysis can be colored by researchers’ pre-understanding of human beings as physical, psychological, and social beings. Other potential limitations of our systematic review should be considered. Even in interview-based surveys, an accurate assessment of experiences surrounding re-traumatization during healthcare is difficult to ensure. The eight studies that contributed to the present review were conducted over several decades, within different research topics and with different samples—including refugees with diverse experiences from different continents and in different host countries. Because our sample studies were few in number, transferring our findings to all survivors of war and torture is not possible.

### Practical implications

Before appointments with survivors, healthcare providers should familiarize themselves with patients’ histories. Such preparation not only helps healthcare providers understand patients’ problems—especially trauma histories—but also shows a genuine interest in patients. Some routine procedures can be extremely stressful for torture survivors, evoking memories of torture. Therefore, preventing or minimizing stressors as much as possible is important. For example, survivors who lack trust may require longer consultations in the process of creating trust. A survivor who was ill-treated by someone in their own cultural group may be more comfortable with a healthcare provider from another background. In some cases, certain procedures—such as small surgical procedures and examinations—should be performed under anesthesia; relaxation, meditation, and other coping strategies can also be helpful in this context. These techniques allow torture survivors to calm themselves when they begin feeling anxious. Importantly, many torture survivors can undergo examinations and procedures without being seriously affected if they are told what the examination or procedure will entail and if they experience empathy. Although some survivors believe that exploring traumatic histories may be re-traumatizing, other survivors expressed that they want their histories to be acknowledged. One of the most important ways for healthcare providers to improve torture survivors’ treatment outcomes is to listen to what they need in order to feel safe. This approach requires that healthcare providers take the time to initiate conversations about war experiences, ask direct questions, and utilize trained interpreters. Acknowledgment from healthcare providers can empower survivors, preventing re-traumatization.

## Conclusion

Survivors of war and torture frequently experience re-traumatization during healthcare encounters. The current review’s framework and findings have provided a foundation for understanding the difficulties that refugees and asylum seekers generally—and torture survivors specifically—experience in receiving somatic healthcare. Subsequently, these findings may help healthcare providers to recognize potential stressors and triggers during healthcare interactions, adapt standard treatments in order to prevent re-traumatization, and create safe treatment environments for torture survivors.

## Supporting information

S1 Table Definitions. (DOCX)

S2 Table Search strategy. (DOCX)

S3 Table The Joanna Briggs Institute checklist for qualitative studies. (DOCX)

S4 Table Summary of qualitative evidence profile. (DOCX)

S1 Fig Data extraction and synthesis. (DOCX)

S1 Checklist PRISMA 2009 checklist. (DOC)

## References

[1] Luci M. and Di Rado D., The Special Needs of Victims of Torture or Serious Violence: A Qualitative Research in EU. Journal of Immigrant & Refugee Studies, 2019: p. 1–16.

[2] Masmas T.N., et al, Asylum seekers in Denmark. Torture, 2008 18(2): p. 77–86. 19289884

[3] Piwowarczyk L., Moreno A., and Grodin M., Health care of torture survivors. JAMA, 2000 284(5): p. 539–541. 10.1001/jama.284.5.539 10918684

[4] Moreno A. and Grodin M., Torture and its neurological sequelae. Spinal Cord, 2002 40(5): p. 213 10.1038/sj.sc.3101284 11987003

[5] Sanders J., Schuman M.W., and Marbella A.M., The epidemiology of torture: a case series of 58 survivors of torture. Forensic science international, 2009 189(1–3): p. e1–e7. 10.1016/j.forsciint.2009.03.026 19428200

[6] Protocol, I., Manual on the effective investigation and documentation of torture and other cruel, inhuman or degrading treatment or punishment. United Nations, 1999.

[7] Gottvall M., Vaez M., and Saboonchi F., Social support attenuates the link between torture exposure and post-traumatic stress disorder among male and female Syrian refugees in Sweden. BMC international health and human rights, 2019 19(1): p. 28 10.1186/s12914-019-0214-6 31488136 PMC6727543

[8] Haagensen J.O., The role of the Istanbul-protocol in the uphill battle for torture survivors being granted asylum in Europe and ensuring the perpetrators pay. Torture, 2007 17(3): p. 238 19289897

[9] Kalt A., et al, Asylum seekers, violence and health: a systematic review of research in high-income host countries. American journal of public health, 2013 103(3): p. e30–e42. 10.2105/AJPH.2012.301136 23327250 PMC3673512

[10] Sigvardsdotter E., et al, Prevalence of torture and other war-related traumatic events in forced migrants: a systematic review. Journal on Rehabilitation of Torture Victims and Prevention of Torture, 2016 26(2): p. 41–73. 27858780

[11] Crosby S.S., et al, Prevalence of torture survivors among foreign-born patients presenting to an urban ambulatory care practice. Journal of general internal medicine, 2006 21(7): p. 764–768. 10.1111/j.1525-1497.2006.00488.x 16808779 PMC1924712

[12] Eisenman D., Keller A., and Kim G., Survivors of torture in a general medical setting: how often have patients been tortured, and how often is it missed? The Western journal of medicine, 2000 172(5): p. 301 10.1136/ewjm.172.5.301 10832420 PMC1070871

[13] Jaranson J.M., et al, Somali and Oromo refugees: correlates of torture and trauma history. American journal of public health, 2004 94(4): p. 591–598. 10.2105/ajph.94.4.591 15054011 PMC1448304

[14] Halvorsen, J.Ø. and N. Sveaass, Psykologi og tortur: Faglige og etiske utfordringer for psykologer sett i lys av FNs torturkonvensjon. Tidsskrift for Norsk Psykologforening, 2009.

[15] Defrin R., Lahav Y., and Solomon Z., Dysfunctional pain modulation in torture survivors: The mediating effect of PTSD. The Journal of Pain, 2017 18(1): p. 1–10. 10.1016/j.jpain.2016.09.005 27687222

[16] Johnson D.R., Burgess T., and Ziersch A.M., I don’t think general practice should be the front line: Experiences of general practitioners working with refugees in South Australia. Australia and New Zealand health policy, 2008 5(1). 10.1186/1743-8462-5-20 18687150 PMC2531177

[17] Schubert C.C. and Punamäki R.-L., Torture and PTSD: prevalence, sequelae, protective factors, and therapy. Comprehensive Guide to Post-Traumatic Stress Disorders, 2016: p. 505–536.

[18] Werbart A., The ‘living dead’—Survivors of torture and psychosis. Psychoanalytic Psychotherapy, 1993 7(2): p. 163–179.

[19] Nordin L. and Perrin S., Pain and Posttraumatic Stress Disorder in refugees who survived torture: The role of pain catastrophizing and trauma‐related beliefs. European journal of pain, 2019 10.1002/ejp.1415 31095807

[20] Weisleder P. and Rublee C., The neuropsychological consequences of armed conflicts and torture. Current neurology and neuroscience reports, 2018 18(3): p. 9 10.1007/s11910-018-0818-6 29445906

[21] Keller A.S., Saul J.M., and Eisenman D.P., Caring for survivors of torture in an urban, municipal hospital. The Journal of ambulatory care management, 1998 21(2): p. 20–9; discussion 43–55. 10.1097/00004479-199804000-00005 10181462

[22] Amris K., Jones L.E., and Williams A.C.d.C., Pain from torture: assessment and management. Pain Reports, 2019 4(6): p. e794 10.1097/PR9.0000000000000794 31984299 PMC6903341

[23] Allodi F.A., Assessment and treatment of torture victims: a critical review. Journal of Nervous and Mental Disease, 1991 10.1097/00005053-199101000-00002 1985148

[24] Bunn M., et al, Group treatment for survivors of torture and severe violence: A literature review. Torture: quarterly journal on rehabilitation of torture victims and prevention of torture, 2015 26(1): p. 45–67. 27857004

[25] Duffy R.M. and Kelly B.D., Psychiatric assessment and treatment of survivors of torture. BJPsych Advances, 2015 21(2): p. 106–115.

[26] Ginzburg, K. and Y. Neria, Mental Health Interventions for Survivors of Torture. Zeitschrift für Psychologie, 2015.

[27] Kazlauskas E., et al, Trauma treatment across Europe: where do we stand now from a perspective of seven countries. European journal of psychotraumatology, 2016 7(1): p. 29450 10.3402/ejpt.v7.29450 26996534 PMC4800285

[28] Amris K. and Williams A.C.d.C., Managing chronic pain in survivors of torture. Pain management, 2015 5(1): p. 5–12. 10.2217/pmt.14.50 25537694

[29] Punamäki R.L., Qouta S.R., and Sarraj E.E., Nature of torture, PTSD, and somatic symptoms among political ex‐prisoners. Journal of traumatic stress, 2010 23(4): p. 532–536. 10.1002/jts.20541 20632392

[30] Murray B. and O’Donnell C., Nursing care in the acute hospital setting: Survivors of torture. Advances in Mental Health, 2013 11(2): p. 188–196.

[31] McColl H., Bhui K., and Jones E., The role of doctors in investigation, prevention and treatment of torture. Journal of the Royal Society of Medicine, 2012 105(11): p. 464–471. 10.1258/JRSM.2012.120100 23257969 PMC3526851

[32] Dallam S.J., A Model of the retraumatization process: a meta-synthesis of childhood sexual abuse survivors’experiences in healthcare. 2010, University of Kansas.

[33] Jennings A., On being invisible in the mental health system. The journal of mental health administration, 1994 21(4): p. 374–387. 10.1007/BF02521356 10138011

[34] Hokland M., Kan noen traumatiserte pasienter ta skade av eksponering for minner om traumer? Tidsskrift-norsk psykologforening, 2006 43(11): p. 1150.

[35] Behnia B., Trust building from the perspective of survivors of war and torture. Social Service Review, 2004 78(1): p. 26–40.

[36] Jacobs U. and Iacopino V., Torture and its consequences: A challenge to clinical neuropsychology. Professional Psychology: Research and Practice, 2001 32(5): p. 458.

[37] Johnson D.R., Helping refugee trauma survivors in the primary care setting. Minneapolis: The Center for the Victims of Torture, 2005.

[38] McCullough-Zander K. and Larson S., ‘The Fear Is Still in Me’: Caring for Survivors of Torture: How to identify, assess, and treat those who have endured this extreme trauma. AJN The American Journal of Nursing, 2004 104(10): p. 54–64. 10.1097/00000446-200410000-00027 15492538

[39] Century G., Leavey G., and Payne H., The experience of working with refugees: Counsellors in primary care. British Journal of Guidance & Counselling, 2007 35(1): p. 23–40.

[40] Rometsch C., et al, Pain, somatic complaints, and subjective concepts of illness in traumatized female refugees who experienced extreme violence by the “Islamic State”(IS). Journal of psychosomatic research, 2020 130: p. 109931 10.1016/j.jpsychores.2020.109931 31981895

[41] Ellis B.H., et al, *Understanding the mental health of refugees*: *Trauma*, *stress*, *and the cultural context*, in *The Massachusetts General Hospital textbook on diversity and cultural sensitivity in mental health*. 2019, Springer p. 253–273.

[42] Kienzler, H., C. Spence, and T. Wenzel, A Culture-Sensitive and Person-Centred Approach: Understanding and Evaluating Cultural Factors, Social Background and History When Working with Refugees. An Uncertain Safety: Integrative Health Care for the 21st Century Refugees, 2018: p. 101.

[43] Kienzler H., Spence C., and Wenzel T., *A Culture-Sensitive and Person-Centred Approach*: *Understanding and Evaluating Cultural Factors*, *Social Background and History When Working with Refugees*, *in An Uncertain Safety*. 2019, Springer p. 101–116.

[44] Van Loenen T., et al, Primary care for refugees and newly arrived migrants in Europe: a qualitative study on health needs, barriers and wishes. The European Journal of Public Health, 2017 28(1): p. 82–87. 10.1093/eurpub/ckx210 29240907

[45] Ahrenholz N.C., Haider M., and Niyogi A., Caring for Refugee and Asylee Torture Survivors in Primary Care. Sgim forum 38 (10), 2015.

[46] Pope K.S., Psychological assessment of torture survivors: Essential steps, avoidable errors, and helpful resources. International journal of law and psychiatry, 2012 35(5–6): p. 418–426. 10.1016/j.ijlp.2012.09.017 23040707

[47] Chester B. and Holtan N., Working with refugee survivors of torture. Western journal of medicine, 1992 157(3): p. 301 1413774 PMC1011282

[48] Eden J., et al, *Standards for Initiating a Systematic Review*, in *Finding What Works in Health Care*: *Standards for Systematic Reviews*. 2011, National Academies Press (US). 24983062

[49] Moher D., et al, Preferred reporting items for systematic review and meta-analysis protocols (PRISMA-P) 2015 statement. Revista Espanola de Nutricion Humana y Dietetica, 2016 20(2): p. 148–160. 10.1186/2046-4053-4-1 PMC4320440 25554246

[50] Morton S., et al, *Finding what works in health care*: *standards for systematic reviews*. 2011: National Academies Press. 24983062

[51] Shamseer L., et al, Preferred reporting items for systematic review and meta-analysis protocols (PRISMA-P) 2015: elaboration and explanation. Bmj, 2015 349: p. g7647 10.1136/bmj.g7647 25555855

[52] Eriksen M.B. and Frandsen T.F., The impact of patient, intervention, comparison, outcome (PICO) as a search strategy tool on literature search quality: a systematic review. Journal of the Medical Library Association: JMLA, 2018 106(4): p. 420 10.5195/jmla.2018.345 30271283 PMC6148624

[53] Dhammi I.K. and Kumar S., *Medical subject headings (MeSH) terms*. 2014, Springer. 10.4103/0019-5413.139827 PMC4175855 25298548

[54] Baumann N., How to use the medical subject headings (Me SH). International journal of clinical practice, 2016 70(2): p. 171–174. 10.1111/ijcp.12767 26763799

[55] Covidence. https://www.covidence.org/reviews/active.

[56] Berman H., Girón E.R.I., and Marroquín A.P., A narrative study of refugee women who have experienced violence in the context of war. Canadian Journal of Nursing Research Archive, 2006 38(4). 17290954

[57] Hermansen M. and Nielsen D., Betingelser i hverdagslivet i eksil, som kan få betydning for mødet med det danske sundhedsvæsen—et kvalitativt studie. Nordisk sygeplejeforskning, 2018 8(03): p. 204–218.

[58] Perron N.J. and Hudelson P., Somatisation: illness perspectives of asylum seeker and refugee patients from the former country of Yugoslavia. BMC Family Practice, 2006 7(1): p. 10. 16480514 10.1186/1471-2296-7-10 PMC1386680

[59] Shannon P., O’Dougherty M., and Mehta E., Refugees’ perspectives on barriers to communication about trauma histories in primary care. Mental health in family medicine, 2012 9(1): p. 47 23277798 PMC3487607

[60] Shannon P.J., Refugees’ advice to physicians: how to ask about mental health. Family Practice, 2014 31(4): p. 462–466. 10.1093/fampra/cmu017 24820520

[61] Fang M.L., et al, Experiencing ‘pathologized presence and normalized absence’; understanding health related experiences and access to health care among Iraqi and Somali asylum seekers, refugees and persons without legal status. BMC public health, 2015 15(1): p. 923 10.1186/s12889-015-2279-z 26386559 PMC4575487

[62] Gruber M. and Byrd R., Post-traumatic stress disorder and GI endoscopy: a case study. Gastroenterology nursing: the official journal of the Society of Gastroenterology Nurses and Associates, 1993 16(1): p. 17–20. 10.1097/00001610-199308000-00005 8399434

[63] Tobin C., Murphy-Lawless J., and Beck C.T., Childbirth in exile: Asylum seeking women’s experience of childbirth in Ireland. Midwifery, 2014 30(7): p. 831–838. 10.1016/j.midw.2013.07.012 24071035

[64] Rowlands B.H., Grounded in practice: Using interpretive research to build theory. The Electronic Journal of Business Research Methodology, 2005 3(1): p. 81–92.

[65] Williams V., Boylan A.-M., and Nunan D., Critical appraisal of qualitative research: necessity, partialities and the issue of bias. BMJ Evidence-Based Medicine, 2020 25(1): p. 9–11. 10.1136/bmjebm-2018-111132 30862711

[66] Briggs, J. https://joannabriggs.org/sites/default/files/201905/JBI_RCTs_Appraisal_tool2017_0.pdf. 2017.

[67] Lewin S., et al, Using qualitative evidence in decision making for health and social interventions: an approach to assess confidence in findings from qualitative evidence syntheses (GRADE-CERQual). PLoS Medicine, 2015 12(10): p. e1001895 10.1371/journal.pmed.1001895 26506244 PMC4624425

[68] Lewin S., et al, Applying GRADE-CERQual to qualitative evidence synthesis findings—paper 2: how to make an overall CERQual assessment of confidence and create a Summary of Qualitative Findings table. Implementation Science, 2018 13(1): p. 10. 29384082 10.1186/s13012-017-0689-2 PMC5791047

[69] Kira I.A., et al, The effects of perceived discrimination and backlash on Iraqi refugees’ mental and physical health. Journal of Muslim Mental Health, 2010 5(1): p. 59–81.

[70] Silove D., et al, Psychosocial needs of torture survivors. Australian & New Zealand Journal of Psychiatry, 1991 25(4): p. 481–490. 10.3109/00048679109064441 1793418

[71] Song S.J., et al, Psychological distress in torture survivors: pre-and post-migration risk factors in a US sample. Social psychiatry and psychiatric epidemiology, 2015 50(4): p. 549–560. 10.1007/s00127-014-0982-1 25403567

[72] Richey S.L., Assessment and management of survivors of torture in the emergency department. Journal of emergency nursing, 2007 33(5): p. 484–487. 10.1016/j.jen.2007.04.018 17884484

[73] Griffiths R., et al, Operation Safe Haven: the needs of nurses caring for refugees. International journal of nursing practice, 2003 9(3): p. 183–190. 10.1046/j.1440-172x.2003.00422.x 12801250

[74] Swahnberg K., et al, Women’s perceived experiences of abuse in the health care system: their relationship to childhood abuse. BJOG: An International Journal of Obstetrics & Gynaecology, 2004 111(12): p. 1429–1436. 10.1111/j.1471-0528.2004.00292.x 15663131

[75] Swahnberg K. and Wijma K., Validation of the abuse screening inventory (ASI). Scandinavian journal of public health, 2007 35(3): p. 330–334. 10.1080/14034940601040759 17530556

[76] Dehghan R., The health impact of (sexual) torture amongst Afghan, Iranian and Kurdish refugees: A literature review. Torture Journal, 2018 28(3): p. 77–91. 10.7146/torture.v28i3.111194 30649843

[77] Schein Y.L., et al, A qualitative study of health experiences of Ethiopian asylum seekers in Norway. BMC Health Services Research, 2019 19(1): p. 958 10.1186/s12913-019-4813-7 31829251 PMC6907115

[78] Engstrom D.W., Roth T., and Hollis J., The use of interpreters by torture treatment providers. Journal of Ethnic & Cultural Diversity in Social Work, 2010 19(1): p. 54–72.

[79] Watson, V.S., Re-Traumatization of Sexual Trauma in Women’s Reproductive Health Care. 2016.

[80] Johnson-Agbakwu C.E., et al, Mental health screening among newly arrived refugees seeking routine obstetric and gynecologic care. Psychological services, 2014 11(4): p. 470 10.1037/a0036400 25383999 PMC4228798

