# Supplementary material for: Uncovering re-traumatization experiences of torture survivors in somatic health care: A qualitative systematic review
Source: PLoS One. 2021 Feb 4;16(2):e0246074. doi: 10.1371/journal.pone.0246074 (PMC7861410; doi:10.1371/journal.pone.0246074)
Supplement: S1 Table — (DOCX) [file pone.0246074.s001.docx]

**Supplemental Table S1: Definitions**

| DESCRIPTION OF THE CONTEXT – SOMATIC HEALTH CARE  The health care delivery setting itself can without intention re-traumatize patients creating a context with challenges for both patients and staff. The context includes any somatic treatment intended to help refugees and asylum seekers who have experienced torture. The relevant interventions to this review include all kind of treatment who intend to improve somatic conditions administrated by health care professionals working in somatic departments. |
| --- |
| ASYLUM SEEKER  An asylum-seeker is a person who has left their country and is seeking protection from persecution and serious human rights violations in another country, but who has not yet been legally recognized as a refugee and is waiting to receive a decision on their asylum claim. Seeking asylum is a human right. This means everyone should be allowed to enter another country to seek asylum (1).    REFUGEE  A refugee is a person who has fled their own country because they are at risk of serious human rights violations and persecution there. The risks to their safety and life were so great that they felt they had no choice but to leave and seek safety outside their country because their own government cannot or will not protect them from those dangers. Refugees have a right to international protection (1). |
| TORTURE  Under the United Nations Torture Convention of 1984, torture involves intentional infliction of pain, by a public official, to obtain information.  The full definition of torture in the convention is "Any act by which severe pain or suffering, whether physical or mental, is intentionally inflicted on a person for such purposes as obtaining from him or a third person information or a confession, punishing him for an act he or a third person has committed or is suspected of having committed, or intimidating or coercing him or a third person, or for any reason based on discrimination of any kind, when such pain or suffering is inflicted by or at the instigation of or with the consent or acquiescence of a public official or other person acting in an official capacity" (2). |
| TORTURE SURVIVORS  The definition of torture survivors in this literature review is consistent with the UN 1984 definition (2). Torture survivors may be found among refugees, asylum seekers, war survivors and survivors of organized violence, in different contexts like prison, refuges camp and detention centres. |
| RE-TRAUMATIZATION  In the current review, we define re-traumatization as “…reactivation of trauma symptoms, memories or feelings related to the torture experience in the past, due to an event or interaction in health care settings that reminds survivors of the previous traumatic torture experiences” (3, 4) |
| References  1. Refugees, Asylum-seekers and migrants [Internet]. Amnesty International. <https://www.amnesty.org/en/what-we-do/refugees-asylum-seekers-and-migrants/>.  2. Protocol I. Manual on the effective investigation and documentation of torture and other cruel, inhuman or degrading treatment or punishment. United Nations. 1999.  3. Dallam SJ. A Model of the Retraumatization Process: A Meta-synthesis of childhood sexual abuse survivors'experiences in healthcare: University of Kansas; 2010.  4. Jennings A. On being invisible in the mental health system. The journal of mental health administration. 1994;21(4):374-87. |
