# Supplementary material for: Uncovering re-traumatization experiences of torture survivors in somatic health care: A qualitative systematic review
Source: PLoS One. 2021 Feb 4;16(2):e0246074. doi: 10.1371/journal.pone.0246074 (PMC7861410; doi:10.1371/journal.pone.0246074)
Supplement: S2 Table — (DOCX) [file pone.0246074.s002.docx]

**Supplemental Table S2: Search Strategy**

| **Databases** | **MEDLINE,**  **Embase,**  **PsycINFO,**  **CINAHL,**  **PILOTS,**  **Web of Science (ISI),**  **WHO: International Clinical Trials Registry Platform /ICTRP),**  **PILOTS (Online database, Published International Literature on Traumatic Stress)**  **Cochrane library of systematic reviews.** |
| --- | --- |
| **Gray Literature** | OPENSIGLE (Online database of reports and other grey literature produced in Europe until 2005),  The Rehabilitation and Research Centre for Torture Victims (https://irct.org/),  The CENTER for VICTIMS of TORTURE (https://www.cvt.org/),  The Canadian center for victims of torture, International Rehabilitation council for torture victims (http://ccvt.org/),  Röda Korsets Center för torterade flyktingar – Stockholm (https://www.redcross.se/rkcstockholm), Dignity – Dansk institute mod tortur (https://dignity.dk/)  Veterans & Veterans Families Counselling Service (VVCS) (https://www.openarms.gov.au/about/vvcs-now-open-arms-veterans-families-counselling) |
| **Search Terms** | Searches in MEDLINE  1. exp Refugees/  2. refugee.tw, kf.  3. (asylum adj5 seeker*).tw, kf.  4. (War adj5 Victim*).tw, kf.  5. exp "Prisoners of War"/  6. (prisoner* adj5 war).tw, kf.  7. exp Torture/  8. (torture adj5 survivor*).tw, kf.  9. (victim* adj5 "ill treatment").tw, kf.  10. exp Refugees/or refugee.tw, kf. or asylum adj5 seeker*.tw, kf. or War adj5 Victim*.tw, kf. or exp "Prisoners of War"/ or prisoner* adj5 war. tw, kf. or exp Torture/ or torture adj5 survivor*.tw, kf. or victim* adj5 "ill treatment").tw, kf.  11. experience*.tw, kf.  12. Perception/  13. perception*.tw, kf.  14. Expectation*.tw, kf.  15. (critical adj5 episode*).tw, kf.  16. obstacle*.tw, kf.  17. opportunities*.tw, kf.  18. Physician-Patient Relations/  19. experience*.tw, kf. or Perception/ or perception*.tw, kf. or Expectation*.tw, kf. or (critical adj5 episode*).tw, kf. or obstacle*.tw, kf. or opportunities*.tw, kf or Physician-Patient Relations/ or Physician-Patient Relations/  20. "Delivery of Health Care"/  21. "health care”. Tw, kf.  22. Primary Health Care/  23. "medical care”. Tw, kf.  24. "somatic car”. Tw, kf.  25. Emergency Service, Hospital/  26. Intensive Care Units/ or Critical Care/  27. Outpatient Clinics, Hospital/ or Hospitalization/  28. General Practice/  29. Family Practice/  30. "Delivery of Health Care"/ or "health car”. Tw, kf. or Primary Health Care/ or "medical care". tw, kf. or "somatic care". tw, kf. or Emergency Service, Hospital/ or Intensive Care Units/ or Critical Care/ or Outpatient Clinics, Hospital/ or Hospitalization/ or General Practice/ or Family Practice/  31. (exp Refugees/or refugee.tw, kf. or asylum adj5 seeker*.tw, kf. or War adj5 Victim*.tw, kf. or exp "Prisoners of War"/ or prisoner* adj5 war.tw, kf. or exp Torture/ or torture adj5 survivor*.tw, kf. or victim* adj5 "ill treatment").tw, kf.) and (experience*. tw, kf. or Perception/ or perception*. tw, kf. or Expectation*. tw, kf. or (critical adj5 episode*).tw, kf. or obstacle*. tw, kf. or opportunities*. tw, kf or Physician-Patient Relations/ or Physician-Patient Relations/) and (. "Delivery of Health Care"/ or "health care". tw, kf. or Primary Health Care/ or "medical care". tw, kf. or "somatic care". tw, kf. or Emergency Service, Hospital/ or Intensive Care Units/ or Critical Care/ or Outpatient Clinics, Hospital/ or Hospitalization/ or General Practice/ or Family Practice/)  32. Stress Disorders, Post-Traumatic/  33. Personal Satisfaction/  34. Attitude to Health/  35. (experience*. tw, kf. or Perception/ or perception*. tw, kf. or Expectation*. tw, kf. or (critical adj5 episode*). tw, kf. or obstacle*. tw, kf. or opportunities*. tw, kf or Physician-Patient Relations/ or Physician-Patient Relations/) or (Personal Satisfaction/) or (Attitude to Health/)  36. (exp Refugees/or refugee. tw, kf. or asylum adj5 seeker*. tw, kf. or War adj5 Victim*. tw, kf. or exp "Prisoners of War"/ or prisoner* adj5 war.tw, kf. or exp Torture/ or torture adj5 survivor*. tw, kf. or victim* adj5 "ill treatment").tw, kf.) or (Stress Disorders, Post-Traumatic/)  37. ("Delivery of Health Care"/ or "health care". tw, kf. or Primary Health Care/ or "medical care". tw, kf. or "somatic care". tw, kf. or Emergency Service, Hospital/ or Intensive Care Units/ or Critical Care/ or Outpatient Clinics, Hospital/ or Hospitalization/ or General Practice/ or Family Practice/) and ((experience*. tw, kf. or Perception/ or perception*. tw, kf. or Expectation*. tw, kf. or (critical adj5 episode*).tw, kf. or obstacle*. tw, kf. or opportunities*. tw, kf or Physician-Patient Relations/ or Physician-Patient Relations/) or (Personal Satisfaction/) or (Attitude to Health/)) and ((exp Refugees/or refugee. tw, kf. or asylum adj5 seeker*. tw, kf. or War adj5 Victim*. tw, kf. or exp "Prisoners of War"/ or prisoner* adj5 war. tw, kf. or exp Torture/ or torture adj5 survivor*. tw, kf. or victim* adj5 "ill treatment").tw, kf.) or (Stress Disorders, Post-Traumatic/)) |
