# Supplementary material for: Uncovering re-traumatization experiences of torture survivors in somatic health care: A qualitative systematic review
Source: PLoS One. 2021 Feb 4;16(2):e0246074. doi: 10.1371/journal.pone.0246074 (PMC7861410; doi:10.1371/journal.pone.0246074)
Supplement: S3 Table — (DOCX) [file pone.0246074.s003.docx]

**Supplemental Table S3: The Joanna Briggs' Checklist for Qualitative Studies**

| **_Author, year_** | **_Is there congruity between the stated philosophical perspective and the research methodology?_** | **_Is there congruity between the research methodology and the research question or objectives?_** | **_Is there congruity between the research methodology and the methods used to collect data?_** | **_Is there congruity between the research methodology and the representation and analysis of data?_** | **_Is there congruity between the research methodology and the interpretation of results?_** | **_Is there a statement locating the researcher culturally or theoretically?_** | **_Is the influence of the researcher on the research, and vice- versa, addressed?_** | **_Are participants, and their voices, adequately represented?_** | **_Is the research ethical according to current criteria or, for recent studies, and is there evidence of ethical approval by an appropriate body?_** | **_Do the conclusions drawn in the research report flow from the analysis, or interpretation, of the data?_** |
| --- | --- | --- | --- | --- | --- | --- | --- | --- | --- | --- |
| **_Berman, 2009_** | _Yes_ | _Yes_ | _Yes_ | _Yes_ | _Yes_ | _Unclear_ | _No_ | _Unclear_ | _Yes_ | _Unclear_ |
| **_Fang, 2015_** | _Unclear_ | _Yes_ | _Yes_ | _Unclear_ | _Yes_ | _Yes_ | _Yes_ | _Unclear_ | _Yes_ | _Yes_ |
| **_Gruber, 1993_** | _Unclear_ | _Unclear_ | _Unclear_ | _Yes_ | _Yes_ | _No_ | _No_ | _Yes_ | _Yes_ | _Yes_ |
| **_Hermansen & Nielsen, 2018_** | _Yes_ | _Yes_ | _Yes_ | _Yes_ | _Yes_ | _Yes_ | _No_ | _Yes_ | _Yes_ | _Yes_ |
| **_Perron & Hudelson, 2006_** | _Unclear_ | _Yes_ | _Yes_ | _Unclear_ | _Yes_ | _Yes_ | _Yes_ | _Unclear_ | _Yes_ | _Yes_ |
| **_Shannon, O'Dougherty, & Mehta, 2012_** | _Unclear_ | _Yes_ | _Yes_ | _Unclear_ | _Yes_ | _No_ | _No_ | _Yes_ | _Yes_ | _Yes_ |
| **_Shannon 2014_** | _Yes_ | _Yes_ | _Yes_ | _Yes_ | _Yes_ | _No_ | _No_ | _Unclear_ | _Yes_ | _Yes_ |
| **_Tobin 2014_** | _Yes_ | _Yes_ | _Yes_ | _Yes_ | _Yes_ | _No_ | _Unclear_ | _Unclear_ | _Yes_ | _Yes_ |

S5: Supplemental table: Summary of Qualitative Evidence Profile
