# Supplementary material for: Uncovering re-traumatization experiences of torture survivors in somatic health care: A qualitative systematic review
Source: PLoS One. 2021 Feb 4;16(2):e0246074. doi: 10.1371/journal.pone.0246074 (PMC7861410; doi:10.1371/journal.pone.0246074)
Supplement: S4 Table — (DOCX) [file pone.0246074.s004.docx]

S4: Supplemental table: Summary of Qualitative Evidence Profile
