# Supplementary material for: Uncovering re-traumatization experiences of torture survivors in somatic health care: A qualitative systematic review
Source: PLoS One. 2021 Feb 4;16(2):e0246074. doi: 10.1371/journal.pone.0246074 (PMC7861410; doi:10.1371/journal.pone.0246074)
Supplement: S1 Fig — (DOCX) [file pone.0246074.s005.docx]

**S1 Fig. Data extraction and synthesis**

Inductive approach

Deductive approach

Gratitude and satisfaction

Health care professionals’ attitudes

Lack of perceived quality in health care

Avoidance

Disempowerment

Invisibility

Silence

Mistrust

**We incorporated the new themes into the priori framework**

(Table 3)

**And produced new conceptual model**

(Figure 2)

**We generated a priori framework using thematic analysis**

Four interactive sub-processes: 1) hypersensitivity to threats to safety, 2) exposure to triggers, 3) post-traumatic stress reactions, and 4) avoidant coping

**We extracted data from included studies**

(Line by line coding relating to refugee experiences)

(Sub-themes)

**We identified relevant studies for analysis**

(Eight studies were included)

**We identified frameworks, conceptual models and theories**

Dallam’s Health Care Re-Traumatization Model

**Research Question**

“How do re-traumatization of torture survivors occur under somatic health care and what are the triggers?”

**We coded evidence from the included studies against a priori framework**

(Grouped the sub-themes in **four main themes)**

**Created new themes by doing thematic analysis on evidence that could not be coded against the framework**

(The fifth theme)
